# Yellow fever: profile of cases and factors associated with death in a hospital in the State of Rio de Janeiro, 2017–2018

**DOI:** 10.11606/s1518-8787.2019053001434

**Published:** 2019-10-16

**Authors:** Claudia Caminha Escosteguy, Alessandra Gonçalves Lisbôa Pereira, Marcio Renan Vinícius Espínola Marques, Tatiana Rodrigues de Araujo Lima, Rafael Mello Galliez, Roberto de Andrade Medronho

**Affiliations:** I Hospital Federal dos Servidores do Estado. Serviço de Epidemiologia. Rio de Janeiro, RJ, Brasil; II Universidade Estácio de Sá. Faculdade de Medicina. Rio de Janeiro, RJ, Brasil; III Universidade do Estado do Rio de Janeiro. Faculdade de Enfermagem. Rio de Janeiro, RJ, Brasil; IV Instituto Estadual de Infectologia São Sebastião. Rio de Janeiro, RJ, Brasil; V Universidade Federal do Rio de Janeiro. Faculdade de Medicina. Rio de Janeiro, RJ, Brasil; VI Universidade Federal do Rio de Janeiro. Instituto de Estudos em Saúde Coletiva. Rio de Janeiro, RJ, Brasil

**Keywords:** Yellow Fever, epidemiology, Hospital Mortality, Risk Factors, Epidemiology, Descriptive

## Abstract

**OBJECTIVE:**

Describe the clinical and epidemiological profile of confirmed cases of yellow fever whose patients were hospitalized in a general hospital for infectious diseases in the State of Rio de Janeiro, Brazil, from March 11, 2017 to June 15, 2018, during a recent outbreak and factors associated with death.

**METHODS:**

This is a retrospective observational study with analysis of secondary databases of local epidemiological surveillance system, and complementary data collection from epidemiological investigation records and clinical records. Study variables included demographic, epidemiological, clinical, and laboratory data. A descriptive statistical analysis and a bivariate and multivariate analysis by logistic regression were performed to analyze factors associated with death.

**RESULTS:**

Fifty-two patients diagnosed with yellow fever were hospitalized, 86.5% male patients, median age 49.5 years, 40.4% rural workers. The most frequent signs and symptoms were fever (90.4%), jaundice (86.5%), nausea and/or vomiting (69.2%), changes in renal excretion (53.8%), bleeding (50%), and abdominal pain (48.1%), with comorbidity in 38.5% of all cases. The lethality rate was 40.4%. Factors significantly associated with a higher chance of death in the bivariate analysis were: bleeding, changes in renal excretion, and maximum values of direct bilirubin, aspartate aminotransferase (AST), alanine aminotransferase (ALT), urea, and creatinine. In the multivariate analysis by logistic regression, only changes in renal excretion and ALT remained significant predictors of higher chance of death. A threshold effect was also observed for AST. The cutoff points identified as high risk for death were ALT > 4,000 U/L and AST > 6,000 U/L.

**CONCLUSIONS:**

This study contributed to the knowledge on the profile of confirmed cases of high severity yellow fever. The main factors associated with death were changes in renal excretion and elevated serum transaminases, especially ALT. High lethality emphasizes the need for early diagnosis and treatment, and the importance of increasing vaccination coverage.

## INTRODUCTION

Yellow fever is an arbovirus of the *Flavivirus* genus from the family *Flaviviridae* . In urban transmission cycle, *Aedes aegypti* is its main vector. In its wild type, it is a zoonosis transmitted in the American continent by vectors *Haemagogus* and *Sabethes* ^1^ . Its severe form is characterized by serious liver injury, with liver and kidney failure that can lead to death. Vaccination is the most important preventive measure^3^ .

The disease is endemic and enzootic in several tropical regions in the Americas and Africa, with periodic outbreaks. In Brazil, cases were described as endemic, particularly in the Amazon region, with sporadic outbreaks outside this area^3^ .

In December 2016, the virus reemerged in the Southeast region in Brazil, starting the largest outbreak of wild yellow fever in recent decades in the country, with 779 cases confirmed in humans in the monitoring period from July 2016 to June 2017, when 262 deaths were reported (lethality rate of 33.6%). The four states of the Southeast region – Minas Gerais (MG), Espírito Santo (ES), Sao Paulo (SP), and Rio de Janeiro (RJ) – reported the highest number of cases. This region has large urban centers with many sites of high density of *Aedes aegypti* infestation, and a large part of local population was not vaccinated against the disease^3 , 4^ .

From July 2017 to May 16, 2018, another major outbreak occurred in the country, with 1,266 cases confirmed in humans and 415 deaths (lethality rate of 32.8%), especially in MG, SP, and RJ^6^ . Deaths were also reported in non-human primates in other Brazilian states, highlighting the importance of adopting measures to reduce the risk of disease reurbanization, including expansion of recommended vaccination areas and fractional dose campaigns^3 , 7^ .

In 2018, according to data of July 5, 2018 from the Rio de Janeiro State Secretary of Health (SES/RJ), 264 cases were confirmed in 32 municipalities, with 85 deaths (32.2%); 13 locations had confirmed epizootic disease, with deaths of monkeys^8^ .

In RJ, the *Instituto Estadual de Infectologia São Sebastião* (IEISS – São Sebastião State Institute of Infectious Diseases), an institute that has been a historical reference for treating infectious diseases, was incorporated into the *Hospital Federal dos Servidores do Estado* (HFSE) on August 20, 2012. The HFSE is a federal general-teaching hospital, which is part of the national network called *Rede de Vigilância Epidemiológica Hospitalar de Interesse Nacional* ^9^ . In this context of yellow fever outbreak, this structure of HFSE/IEISS played an important role in providing care to severe cases, hospitalizing 52 patients from March 11, 2017 to June 15, 2018. This study aimed to describe the clinical and epidemiological profile of these cases and the factors associated with death.

## METHODS

This study was conducted in a reference hospital for infectious diseases in Rio de Janeiro; it offers an epidemiology service that is part of the national network, which actively searches the cases treated in that unit^9^ . Rio de Janeiro boards the states of MG, ES and SP, and the Atlantic Ocean, covering 43,781,588 km^2^. It has 92 municipalities and 16,718,956 inhabitants (according to 2017 data), 96.7% of them living in urban areas^10^ . About 30% of the state area is covered by the Atlantic Forest^11^ .

From March 11, 2017 to June 15, 2018, 68 cases of suspected yellow fever were reported to the HFSE/IEISS, according to the definition of epidemiological surveillance: “individual with acute fever (up to seven days) of sudden onset, accompanied by jaundice or bleeding, who lives in or has visited a risk area for yellow fever or sites with confirmed epizootic disease in non-human primates or virus isolation in mosquito vectors for the past 15 days, not vaccinated against yellow fever or presenting unknown vaccination status”^1 , 2^ . Of these, 52 cases were confirmed and hospitalized (43 in 2018), constituting the study sample. The disease was confirmed by polymerase chain reaction (PCR) test in 51 cases, and one case was confirmed by epidemiological link^1 , 2^ .

This is a retrospective observational study with analysis of secondary databases of the HFSE epidemiological surveillance system from Sinan – disease reporting system, and complementary data collection from clinical records by the epidemiology service team during the epidemiological investigation process. Demographic, epidemiological, clinical and laboratory variables were analyzed in a spreadsheet-based database exported from Sinan and complemented by typing additional variables to those existing in the investigation form for yellow fever^2 , 12^ , corresponding to signs, symptoms and laboratory tests (detailed in Table 1 and Table 2).

**Table 1:** Profile of 52 confirmed cases of yellow fever whose patients were hospitalized in HFSE/IEISS. Rio de Janeiro, RJ, Brazil, March 11, 2017 to June 15, 2018.

Clinical data	f	%
Male	45	86.5
Age (years)		
13–34	12	23.1
35–49	14	26.9
50–59	13	25.0
60–75	13	25.0
Fever	47	90.4
Jaundice	45	86.5
Nausea/vomiting	36	69.2
Headache	28	53.8
Changes in renal excretion (oliguria, anuria or both)	28	53.8
Bleeding	26	50.0
Abdominal pain	25	48.1
Myalgia	17	32.7
Prostration/asthenia	14	26.9
Diarrhea	9	17.3
Choluria	9	17.3
Arthralgia	6	11.5
Seizures	6	11.5
Retro-orbital pain	2	3.8
Articular edema	2	3.8
Rash	1	1.9
Faget sign	1	1.9
Conjunctival hyperemia	1	1.9
Thrombocytopenia < 100,000/mm^3^	45	86.5
Leucopenia	34	65.4
Comorbidity (at least one)	20	38.5
Arterial hypertension	10	19.2
Alcoholism	9	17.3
Diabetes	5	9.6
Smoking	4	7.7
Hepatic steatosis	2	3.8
Rheumatoid arthritis	2	3.8
Epidemiological history and conclusion of investigation		
Presence of *Aedes aegypti* in the region	51	98.1
Epizootic diseases in the municipality of patient’s residence	35	67.3
Vaccinated against yellow fever	9	17.3
Final classification as wild yellow fever	52	100.0
Laboratory confirmation criterion (PCR)	51	98.1
Autochthonous case of the municipality of patient’s residence	34	65.4
Occupational disease	10	19.2
Outcome		
Death due to wild yellow fever	21	40.4
Healed patient	31	59.6

HFSE/IEISS: *Hospital Federal dos Servidores do Estado* / *Instituto Estadual de Infectologia São Sebastião* ; PCR: polymerase chain reaction.

**Table 2:** Distribution of serum values of some exams according to the evolution of confirmed cases of yellow fever admitted to HFSE/IEISS. Rio de Janeiro, RJ, Brazil, March 11, 2017 to June 15, 2018.

Serum values	Total cases		Death		Non-death		Mann-Whitney test (p)
		
	n	Median (min.–max.)	n	Median (min.–max.)	n	Median (min.–max.)	
Max. AST (U/L)	50	4,833 (69–12,970)	19	8,460 (1,803–12,970)	31	1,820 (69–10,900)	0.000
Max. ALT (U/L)	51	2,920 (86–8,064)	20	5,379 (1,943–8,064)	31	1,581(86–5,428)	0.000
Max. TB (mg/dL)	52	5.62 (0,70–38.00)	21	7.00 (2.00–38.00)	31	4.23 (0.70–29.00)	0.063
Max. DB (mg/dL)	51	4.03 (0.09–20.00)	21	5.00 (2.00–20.00)	30	3.07 (0.09–17.00)	0.043
Max. urea (mg/dL)	49	54 (15–180)	20	93 (45–161)	29	36 (15–180)	0.000
Max. creatinine (mg/dL)	52	1.72 (0.7–17.5)	21	3.2 (0.80–11.7)	31	1.2 (0.7–17.5)	0.000
Min. platelet (per mm^3^)	52	50,000 (10,000–232,000)	21	38,000 (10,000–26,000)	31	68,000 (15,000–232,000)	0.008
Min. hematocrit (%)	50	36.0 (12.9–51.6)	20	29.5 (13.2–51.6)	30	38.5 (12.9–50.0)	0.081

HFSE/IEISS: *Hospital Federal dos Servidores do Estado* / *Instituto Estadual de Infectologia São Sebastião* ; AST: aspartate aminotransferase; ALT: alanine aminotransferase; TB: total bilirubin; DB: direct bilirubin.

Statistical analysis was performed in SPSS v.18 software, including descriptive statistical analysis and a bivariate and multivariate analysis by logistic regression to study the association of independent variables with the outcome of death. Odds ratio (OR) with 95% confidence intervals (95%CI) was used to estimate the chance of death.

In bivariate analysis, Pearson’s chi-square test or Fisher’s exact test was used for categorical variables, and Mann-Whitney test for continuous variables, with p value < 0.05 considered statistically significant. K-means clustering analysis was used to identify the cutoff point for numerical variables corresponding to laboratory test results; it consists of dividing data into a predefined number of clusters. This type of analysis is used when there is no *a priori* hypothesis about the structure or behavior of data; it constitutes a non-hierarchical cluster by partition. Data from each continuous variable were divided into two clusters, starting with the identification of minimum and maximum values recorded. At the end of the process, a cutoff point was obtained between the mean of the maximum value of the first cluster and the minimum value of the second cluster.

In the analysis by logistic regression, variables associated with death in the previous bivariate analysis, with p < 0.10, were included, as well as those variables considered of clinical importance (such as age, sex, comorbidity, and abdominal pain) or of interest (such as year the disease was reported). The Wald test analyzed the significance of independent variables included in the study. For global adjustment analysis, the likelihood ratio test (-2 log likelihood) was used. The agreement between probabilities predicted by the models and the responses obtained were analyzed using the Hosmer-Lemeshow test.

This study was included in a project approved by the HFSE Research Ethics Committee (protocol CEP-HFSE 000.534 of July 14, 2014).

## RESULTS

**Figure 1:**
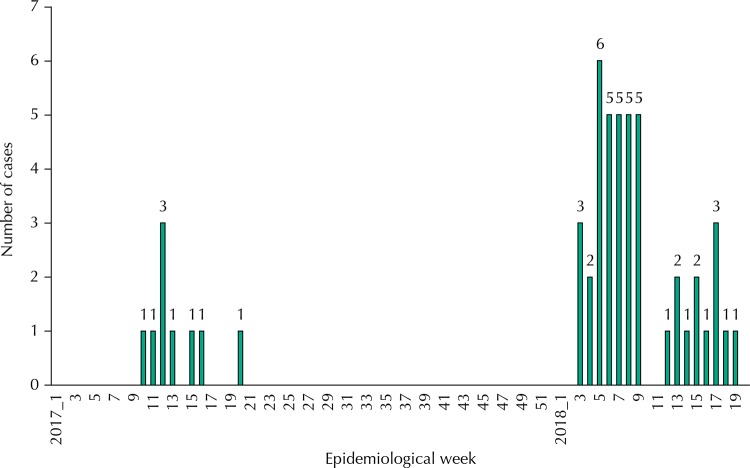
Distribution according to epidemiological week for reporting of confirmed cases of yellow fever admitted to the *Hospital Federal dos Servidores do Estado/Instituto Estadual de Infectologia São Sebastião* . Rio de Janeiro, RJ, Brazil, March 11, 2017 to June 15, 2018.

Figure 1 shows the temporal distribution of cases by epidemiological week of disease reporting; 35 (78.8%) occurred from January to March 2018. Of these, 45 (86.5%) were male patients and seven (13.5%) were female patients, aged 13 to 75 years (median 49.5 years). Self-reported race or skin color was white in 29 (55.8%), brown in 15 (28.8%), black in seven (13.5%), and yellow in one case (1.9%); 27 patients had incomplete basic education (73.0%; excluding 15 of unknown education).

Yellow fever cases were reported in 18 municipalities of RJ, 50 residents and two Chilean tourists. In 2018, 21 patients came from the tourist region of Costa Verde, 18 from Baía de Ilha Grande (16 residents and the two Chilean tourists). The area of residence or accommodation was rural in 32 (61.5%), urban in 11 (21.2%), peri-urban in two (3.8%), and unknown in seven (13.5%) cases. Rural workers prevailed (21 cases; 40.4%); three patients were from tourism (two tourists and one guide).

Table 1 shows the profile of cases according to clinical and epidemiological variables, and the conclusion of the epidemiological investigation. The most frequent signs and symptoms were fever (90.4%), jaundice (86.5%), and nausea and vomiting (69.2%). Changes in renal excretion (oliguria or anuria) occurred in 53.8%, bleeding in 50.0%, and abdominal pain in 48.1% of the cases. Thrombocytopenia < 100,000/mm^3^ occurred in 86.5% and leukopenia in 65.4% of the cases.

At least one comorbidity was described in 38.5% of the cases; the most frequent ones were: systemic arterial hypertension (19.2% of cases), alcoholism (17.3%), and diabetes (9.6%). *Aedes aegypti* was reported in 98.1% of the cases in an urban area where the patient visited. Epizootic diseases in non-human primates were reported in the place of residence in 67.3% of the patients.

Nine cases (17.5%) reported history of previous vaccination against yellow fever; the period between the vaccination date and the onset of symptoms was up to four days in five patients; between 11 and 12 days in two patients; seven years in one patient; and one patient was vaccinated on the third day after symptom onset. In cases of recent vaccination, wild-type virus was confirmed by sequencing, ruling out any post-vaccination adverse event.

In 65.4% of the cases, the transmission was autochthonous of the municipality of residence; in the other cases, it was unknown, due to travels to other municipalities in the state. In 19.2%, the occupational bond was well characterized ( Table 1 ). Regarding disease progression, 21 cases (40.4%) died (19 in 2018 and 2 in 2017).

The period between symptom onset and admission to HFSE/IEISS ranged from one to 15 days, median of 5 days, with similar periods when comparing cases leading and not leading to death. The time between symptom onset and death ranged from four to 22 days, median of 9 days. Hospitalization period ranged from one to 56 days, median of 10 days, with no significant difference when comparing cases of death (1 to 26 days; median = 11) and survivors (3 to 56 days; median = 10). The period between admission and case being reported to the epidemiological surveillance system ranged from zero to three days, median of one; 86.5% were reported up to 24 hours after admission.

Table 2 shows the distribution of maximum serum values of aspartate aminotransferase (AST), alanine aminotransferase (ALT), total bilirubin (TB), direct bilirubin (DB), urea, creatinine, and the lower values of platelet count and hematocrit for the group of cases and according to the outcome of death. A significant association was observed between higher AST, ALT, DB, urea, and creatinine values and death, and between lower values of platelet count and death.

According to Table 3 , the presence of the following signs and symptoms was significantly associated with a higher chance of death: jaundice, bleeding, changes in renal excretion, and seizures. Leukopenia was significantly associated with lower lethality. The cutoff points for AST, ALT, urea, and creatinine were identified by K-means analysis, which defined two clusters (1 and 2) for each one, with minimum and maximum values described as follows: AST – cluster 1 (69 and 5,680 U/L) and 2 (6,260 and 12,970 U/L); ALT – 1 (86 and 3,846 U/L) and 2 (4,160 and 8,064 U/L); urea – 1 (15 and 93 mg/dL) and 2 (110 and 180 mg/dL); creatinine – 1 (0.7 and 5.7 mg/dL) and 2 (7 and 17.5 mg/dL). The following cutoff points of serum laboratory results were significantly associated with higher chance of death: AST > 6,000 U/L; ALT > 4,000 U/L; urea > 100 mg/dL; and creatinine > 6 mg/dL. A subgroup of 20 cases of maximum AST values below 3,000 U/L had 5.0% lethality (one death), and another subgroup of 18 cases of ALT values below 3,500 U/L presented 5.6% lethality (one death). No association was observed between death and the variables of sex, age, race or skin color, education, year of disease reporting, delayed onset of symptoms and hospital admission, abdominal pain, and comorbidity.

**Table 3:** Lethality and estimated chance of death according to some variables of confirmed cases of yellow fever admitted to HFSE/IEISS. Rio de Janeiro, RJ, Brazil, March 11, 2017 to June 15, 2018.

Clinical variables and serum laboratory exams	Present			Absent			Chi-square test (p)	Odds ratio	95%CI
	
	Cases	Deaths		Cases	Deaths				
			
	f	f	%	f	f	%			
Jaundice	45	21	46.7	7	0	0.0	0.033^a^	NA	NA
Bleeding	26	17	65.4	26	4	15.4	0.000	10.389	2.728–39.560
Changes in renal excretion	28	19	67.9	24	2	8.3	0.000	23.222	4.457–120.989
Seizures	6	5	83.3	46	16	34.8	0.034^a^	9.375	1.007–87.284
Leucopenia	34	10	29.4	18	11	61.1	0.027	0.265	0.079–0.881
Max. AST^b^ > 6,000 U/L	20	14	70.0	30	5	16.7	0.000	11.667	3.009–45.239
Max. ALT^c^ > 4,000 U/L	16	13	81.3	35	7	20.0	0.000	17.333	3.852–77.994
Max. urea > 100 mg/dL	12	9	75.0	37	11	29.7	0.006	7.091	1.607–31.296
Max. creatinine > 6 mg/dL	12	8	66.7	40	13	32.5	0.034	4.154	1.055–16.355

HFSE/IEISS: *Hospital Federal dos Servidores do Estado* / *Instituto Estadual de Infectologia São Sebastião* ; NA: not evaluated.

^a^ Fisher’s exact test.

^b^ Aspartate aminotransferase.

^c^ Alanine aminotransferase.

Some logistic models were studied in the multivariate analysis, starting with a saturated model with sex, age, comorbidities, bleeding, changes in renal excretion, leukopenia and continuous laboratory variables (bilirubin, AST, ALT, urea, creatinine and platelet count), using the stepwise method. Model 1 ( Table 4 ) kept a significant association with higher chance of death, changes in renal excretion, and increasing ALT values. Models 2 and 3 were built by the enter method, keeping the AST value, although it did not present statistical significance. These models kept the association of changes in renal excretion with higher chance of death. In addition, in model 2, where transaminases were categorized into ranges identified in the bivariate analysis (ALT ≤ 4,000 U/L and ALT > 4,000 U/L; AST ≤ 6,000 U/L; AST > 6,000 U/L), a significant association was found between higher chance of death and ALT > 4,000 U/L, and borderline significance for AST > 6,000 U/L. In model 3, transaminases vary every 1,000 U/L. At every increase of 1,000 U/L in ALT, a significant increase of 137% is observed in the chance of death, and an increase in borderline significance for AST. Models 1, 2, and 3 showed satisfactory agreements (85.7%, 88.0%, and 90.0%, respectively). The Hosmer-Lemeshow test confirmed satisfactory adjustments for all three models, obtaining non-significant values for the difference between expected and observed frequencies in each decile of estimated probability of death (p = 0.490, p = 0.179, and p = 0.799, respectively).

**Table 4:** Multivariate analysis by logistic regression of factors associated with death in confirmed cases of yellow fever admitted to HFSE/IEISS. Rio de Janeiro, RJ, Brazil, March 11, 2017 to June 15, 2018.

Variable	Parameter	Standard error	p	Odds ratio	95%CI
Model 1 (agreement: 85.7%)^a^					
Intercept	-6.504	2.063	0	-	-
Changes in renal excretion (reference: absent)	3.314	1.29	0.01	27.501	2.193–344.82
ALT (continuous)	0.001	0	0.004	1.001	1–1.002
Model 2 (agreement: 88.0%)^b^					
Intercept	-4.48	1.401	0.001	-	-
Changes in renal excretion (reference: absent)	3.433	1.261	0.006	30.977	2.618–366.563
ALT > 4,000 U/L (reference: ≤4,000 U/L)	2.811	1.165	0.016	16.633	1.696–163.122
AST > 6,000 U/L (reference: ≤6,000 U/L)	1.7	0.926	0.066	5.471	0.891–33.582
Model 3 (agreement: 90.0%)^c^					
Intercept	-8.101	2.773	0.003	-	-
Changes in renal excretion (reference: absent)	3.218	1.413	0.023	24.981	1.567–398.245
ALT 1,000 (variation at every 1,000 U/L)	0.862	0.399	0.031	2.367	1.083–5.177
AST 1,000 (variation at every 1,000 U/L)	0.394	0.226	0.081	1.483	0.953–2.310

HFSE/IEISS: *Hospital Federal dos Servidores do Estado* and *Instituto Estadual de Infectologia São Sebastião*

^a^ Model 1: stepwise forward, based on sex, age, comorbidity, changes in renal excretion, aspartate aminotransferase (AST), alanine aminotransferase (ALT), direct bilirubin, urea, creatinine, and leucopenia.

^b^ Model 2: enter, with categorical ALT and AST.

^c^ Model 3: enter, with ALT and AST on a scale of 1,000 units of variation.

Regarding the treatment, in addition to intensive support measures, high-volume plasma exchange was performed for removal of plasma cytokines and adhesion molecules and for coagulation factor replacement in 12 patients (23.1%), with 5 deaths (41.7%); lethality was 40.0% in remaining cases. Hemodialysis was performed in 21 cases (40.4%), with 16 deaths (lethality of 76.2% versus 16.1% in remaining cases; OR = 16.6; 95%CI 4.2–66.2). In a multivariate model controlling transaminases, urea, creatinine, and platelet count, the association between hemodialysis and death lost statistical significance (OR = 8.3; 95%CI 0.6–119.2).

## DISCUSSION

This study presented the profile of 52 confirmed cases of yellow fever whose patients were admitted to HFSE/IEISS, the hospital in charge of 17.9% of all cases in the State of Rio de Janeiro in 2017 and 2018. In 2017, SES/RJ confirmed 27 cases with 9 deaths^13^ ; in 2018, 264 cases and 85 deaths^8^ . The temporal distribution of hospitalized patients followed the dynamics of the outbreak in the state.

Male predominance is consistent with the literature^14^ . Median age (49.5 years) was higher than other studies: 26 and 27.5 years^15 , 16^ . Case studies in the extra-Amazon region have reported median age closer to this study, such as median age of 36 years^14^ and mean age of 46.7 years^18^ . Differences in the distribution of cases of wild yellow fever by age and sex vary according to the degree of exposure to the woods^14^ .

The rural area of residence was observed in 61.5% of the cases, similar to another study^14^ . Regarding patient occupation, 40.4% were rural workers with exposure to the wild type of yellow fever; other studies report similar or higher percentages, from 41.7%^17^ to 79.2%^14 , 16 , 18^ . The contribution of tourism activity has been described^14 , 17^ . A recent report showed 10 cases of yellow fever among travelers in Brazil, with four deaths; three were Chilean travelers, of these, two are included in this study^21^ .

Regarding vaccination status, in a study that analyzed 281 confirmed cases from 1999 to 2003, of which 37.4% were in the Amazon, 90.8% were unvaccinated and the remaining 9.2% received only one dose^14^ . In that study, individuals who received the vaccine up to ten days before the onset of symptoms were considered unvaccinated^4^ . Based on this criterion, only three cases of the present study would be considered effectively vaccinated. In the context of this study, excluding the differential diagnosis with adverse event after vaccination became relevant in cases of recent vaccination.

According to estimates, at least 90% of yellow fever cases are asymptomatic or oligosymptomatic, and 10% are severe forms associated with high lethality.^5^ Frequently reported symptoms are fever (85%^18^ to 94.4%^15 , 19^ ), headache (70.3%^18^ to 83.3%^15 , 19^ ), myalgia (27.8%^19^ to 76.5%^18^ ), and vomiting (69.4%^19^ to 75.8%^15^ ), in percentages that are similar to those found in this study – except for headache, which was lower (53.8%).

However, the percentage found in this study for jaundice (86.5%) is higher than in other investigations of 35.5% to 69.1%^15 , 18 , 19^ . Also, changes in renal excretion (53.8%) are higher than reported by other authors: 16.7%^19^ to 36.7%^15^ . The latter reported bleeding at 46.4%, a percentage close to this study (50%).

According to the literature, around 15% of cases develop a visceral disease with jaundice. In this situation, the lethality rates range from 20% to 50%^4 , 22^ . The lethality rate observed in Africa has been around 20%, generally lower than in South America (40%–60%)^4^ . The lethality rates reported in patients with severe viscerotropic or post-vaccination disease are high, above 50%, even in developed intensive care centers^22^ .

In Brazil, the lethality rate of severe cases varies between 40 and 60%^5 , 14^ . Some studies report lower lethality rates, but with different severity profile. An outbreak in the State of Maranhão, with asymptomatic cases accounting for 40.0%, reported lethality rate of 17.5%^20^ . A recent study conducted in Minas Gerais found a lethality rate of 13.5%, but the frequency of jaundice was 35.5%^18^ , lower than this study (86.5%). An outbreak in MG, when 69.4% of patients were hospitalized, reported a lethality rate of 33.3%; in 19 cases classified as severe (with bleeding, liver and kidney failure, severe prostration, or coma), the lethality rate was 83.3%^19^ .

Based on the jaundice criterion only, 86.5% of the cases in this study would be of moderate or severe clinical form^1 , 4^ . Another study described 70% of moderate to severe cases, 69.1% jaundice, and 44.2% deaths^15^ .

No association was observed between death and sex, age, race or skin color, education, year of disease reporting, period between symptom onset and hospital admission, abdominal pain, and comorbidity. A study confirmed cases in Brazil from 2000 to 2012^16^ found higher lethality rates among male patients (49.6%) than among female patients (39.7%), but the difference was not significant. A study analyzing cases from a previous period (1999–2002) found a significant association between higher chance of death and male patients and age over 40 years, but in the multivariate analysis, sex and age lost significance^15^ .

The median interval between symptom onset and admission to HFSE/IESS (five days) was slightly longer than another study (three days)^19^ . Both studies found no difference between this interval and death, which is in agreement with at least another study^18^ . In this study, the patients went first to other units, with subsequent referral to HFSE/IEISS.

A study conducted in MG^18^ reported a shorter hospitalization period than this study, and no difference regarding the outcome (mean = 5.70 days and SD = 3.78 days in the case of death; mean = 5.02 days and SD = 5.88 days for the survivors). Another study reported median hospitalization period until death of eight days^19^ , shorter than this study. The severity of the cases analyzed in this study and the use of intensive care, hemodialysis and high-volume plasma exchange are some factors possibly related to longer hospitalization period and high cost of care.

In general, the serum values of transaminases, bilirubin, urea and creatinine in this study were higher than those reported in the literature. A study analyzing 36 cases, 91.7% of severe and moderate forms^19^ , described for AST 47 to 7,500 U/L (mean = 1,027.8); ALT 26 to 6,600 U/L (mean = 815.8); DB 0.2 to 12.4 mg/dL (mean = 3.2); urea 10 to 175 mg/dL (mean = 56.5); creatinine 0.6 to 13 mg/dL (mean = 2.9). Another study^15^ analyzing 251 cases, 70.4% of moderate to severe forms, reported lower median values of transaminases, although the maximum values were higher than those found in this study: AST median 1,096 U/L (ranging from 13 to 43,460); ALT median 957 U/L (9 to 15,250); DB median 4.7 mg/dL (0.4 to 77.0). However, for urea and creatinine, their values were higher: urea median 106 mg/dL (11 to 328), and creatinine median 3.6 mg/dL (1.0 to 19.0).

In this and in the two other studies^15 , 19^ , AST increases generally exceeded those of ALT, which is described in the literature^5 , 23^ probably due to the cytopathic effect of the virus on the myocardium and skeletal muscles; ALT is more specific for liver disease than AST. Regarding the increased bilirubin level, it was mainly due to the increase in direct-reacting fraction, in agreement with the literature^5^ .

In this study, a significant association was found between higher AST, ALT, DB, urea and creatinine values and death, and between lower platelet count and death values. In the multivariate analysis, only changes in renal excretion and ALT remained significantly associated with higher chance of death; AST maintained an association with threshold p value. Another study^15^ found an association with higher chance of death for male patients, age > 40 years, jaundice, AST > 1,200 U/L, ALT > 1,500 U/L, TB > 7 mg/dL, DB > 5 mg/dL, and urea > 100 mg/dL. However, the multivariate analysis showed associations only with jaundice and AST > 1,200 U/L.

No specific treatment has been defined so far. Hospitalization of severe forms is recommended in hospitals that offer intensive care and dialysis^1 , 2 , 23^ . Some antiviral drugs have been evaluated, such as ribavirin, thiazofurine, carboxamide, and pyrazoline compounds^4 , 22^ , and more recently, sofosbuvir^7^ . Catastrophic terminal events associated with severe cases, possibly related to a systemic inflammatory response syndrome, represent a potential field for evaluating the role of interventions such as high-volume plasma exchange^7^ , which was performed at the institution. It was based on the logic of fulminant hepatitis management guidelines; the protocol used was that reported by Larsen et al.^24^ , adapted to the circumstances of yellow fever outbreak.

Liver transplantation in fulminant hepatitis attributed to yellow fever was first performed in December 2017 in São Paulo, and its role was not well clarified^7 , 23 , 25^ . In this study, one case was transferred to another unit for liver transplantation, progressing to death less than 24 hours after the transfer during the transplant surgery (death included in this study).

Reemergence of yellow fever outside the Amazon region reached regions near large urban centers, and a large part of local population was not vaccinated. In the last two years, the disease has disseminated, particularly in the Southeast region, resulting in the largest epidemic in recent decades. The causes of this expansion have not been clearly explained; environmental factors and alterations of the virus itself have been investigated^7^ . In 2017, a mutation in viral replication enzymes was identified, but its impact has not been fully explained^26^ . A recent study reported the yellow fever virus responsible for outbreaks from 2016 to 2018 in the Southeast region in Brazil belongs to a monophyletic lineage (BR-YFV_2016/18), probably coming from the Midwest to the Southeast. Its persistence in the Southeast suggests that this region has proper ecological and climatic conditions for virus survival during epidemic and interepidemic periods^27^ . Described factors include extended rainy season associated with abundant vectors, deforestation for agriculture, epizootic transmission, and displacement of individuals in virus period^4 , 28^ .

High infestation of *Aedes aegypti* in several urban centers of these regions, increased number of wild yellow fever cases, and low vaccination coverage are factors that favor the risk of disease reurbanization. Recent outbreaks of wild yellow fever starting in December 2016 in the states of MG, ES, RJ and SP, and confirmed deaths of monkeys in other states have once again emphasized the relevance of adopting measures to reduce the risk of reurbanization, with changes in vaccination recommendations, including expansion of recommended vaccination areas and fractional dose campaigns^3 , 7^ .

In this context, this study presented the experience of a hospital that significantly contributed to the treatment of severe cases of yellow fever in the state. The severity of the cases is represented by the high percentage of jaundice (86.5%), changes in renal excretion (53.8%), and bleeding (50.0%), besides the higher median values of serum transaminases. Providing care to these patients involves a highly complex structure adapted to respond to emergencies and representing a great challenge for the health team. A deeper knowledge about the profile of these cases, especially the factors associated with higher chance of death, may contribute to discussions regarding the role of therapeutic interventions such as high-volume plasma exchange. Study limitations are mainly related to the quality of information from the epidemiological surveillance system, which was minimized after the review, and data complementation with medical records during the epidemiological investigation and case end process, which is part of the epidemiology service routine^9^ . Missing information was less than 5% for most of the analyzed variables, except for education (15.0%) and maximum urea (7.7%), and much lower when compared to other studies^15^ .

Active epidemiological surveillance contributed to fast reporting to health authorities, monitoring of sample collection to confirm the cases, and the maintenance of an information system that, even during an outbreak, was able to support this study. The study contributed to a better understanding of the profile of yellow fever cases in HFSE/IEISS, which admitted 17.9% of the confirmed cases in RJ in this period, showing a high severity profile. The main predictors of death were changes in renal excretion and elevated serum transaminases, especially ALT. High lethality emphasizes the need for early diagnosis and treatment, and the importance of increasing vaccination coverage.
