# Supplementary material for: Yellow fever: profile of cases and factors associated with death in a hospital in the State of Rio de Janeiro, 2017–2018
Source: Rev Saude Publica. 2019 Oct 16;53:89. doi: 10.11606/s1518-8787.2019053001434 (PMC6802947; doi:10.11606/s1518-8787.2019053001434)
Supplement: Translation

# Translation: Febre amarela: perfil dos casos e fatores associados ao óbito em hospital referência no estado do Rio de Janeiro, 2017–2018

**Authors:** Claudia Caminha Escosteguy, Alessandra Gonçalves Lisbôa Pereira, Marcio Renan Vinícius Espínola Marques, Tatiana Rodrigues de Araujo Lima, Rafael Mello Galliez, Roberto de Andrade Medronho

**Affiliations:** I Hospital Federal dos Servidores do Estado. Serviço de Epidemiologia. Rio de Janeiro, RJ, Brasil; II Universidade Estácio de Sá. Faculdade de Medicina. Rio de Janeiro, RJ, Brasil; III Universidade do Estado do Rio de Janeiro. Faculdade de Enfermagem. Rio de Janeiro, RJ, Brasil; IV Instituto Estadual de Infectologia São Sebastião. Rio de Janeiro, RJ, Brasil; V Universidade Federal do Rio de Janeiro. Faculdade de Medicina. Rio de Janeiro, RJ, Brasil; VI Universidade Federal do Rio de Janeiro. Instituto de Estudos em Saúde Coletiva. Rio de Janeiro, RJ, Brasil

**Keywords:** Febre Amarela, epidemiologia, Mortalidade Hospitalar, Fatores de Risco, Epidemiologia Descritiva

## Abstract

**OBJETIVO:**

Descrever o perfil clínico-epidemiológico dos casos confirmados de febre amarela internados em hospital geral de referência para doenças infecciosas no estado do Rio de Janeiro, Brasil, de 11 de março de 2017 a 15 de junho de 2018, durante recente surto e fatores associados ao óbito.

**MÉTODOS:**

Estudo observacional retrospectivo, com análise de bases de dados secundários da vigilância epidemiológica local e coleta complementar de dados nas fichas de investigação epidemiológica e prontuários clínicos. As variáveis analisadas incluíram dados demográficos, epidemiológicos, clínicos e laboratoriais. Foi conduzida análise estatística descritiva bivariada e múltipla por regressão logística para estudo de fatores associados ao óbito.

**RESULTADOS:**

Foram internados 52 casos confirmados, 86,5% deles homens, com mediana de idade de 49,5 anos e 40,4% trabalhadores rurais. Os sinais e sintomas mais frequentes foram: febre (90,4%), icterícia (86,5%), náuseas e/ou vômitos (69,2%), alterações de excreção renal (53,8%), hemorragias (50%) e dor abdominal (48,1%), com comorbidade em 38,5% dos casos. A letalidade foi de 40,4%. Os fatores associados significativamente à maior chance de óbito na análise bivariada foram: hemorragia, alterações de excreção renal e valores máximos de bilirrubina direta, aspartato aminotransferase (AST), alanina aminotransferase (ALT), ureia e creatinina. Na análise múltipla por regressão logística, apenas alterações de excreção renal e ALT permaneceram como preditores significativos de maior chance de óbito. Observou-se ainda efeito limítrofe para AST. Os pontos de corte identificados como de alto risco para óbito foram ALT > 4.000 U/L e AST > 6.000 U/L.

**CONCLUSÕES:**

O estudo contribuiu para o conhecimento do perfil de casos confirmados de febre amarela com gravidade alta. Os principais fatores associados ao óbito foram a alteração da excreção renal e a elevação sérica de transaminases, sobretudo a ALT. A letalidade elevada reforça a necessidade de diagnóstico e tratamento precoces, e a importância do incremento da cobertura vacinal.

## INTRODUÇÃO

A febre amarela é uma arbovirose produzida por um *Flavivirus* , família *Flaviviridae.* No ciclo de transmissão urbana, o *Aedes aegypti* é seu principal vetor. No ciclo silvestre, é uma zoonose transmitida no continente americano pelos vetores *Haemagogus* e *Sabethes* ^1^. A forma grave caracteriza-se por lesão hepática importante, apresentando manifestações de insuficiência hepática e renal que podem levar ao óbito. A vacinação é a medida preventiva mais importante^3^.

A doença é endêmica e enzoótica em diversas regiões tropicais das Américas e da África, com a ocorrência de surtos periódicos. No Brasil, eram descritos casos de forma endêmica, principalmente na região amazônica, com surtos esporádicos fora dessa área^3^.

Em dezembro de 2016, o vírus reemergiu no sudeste brasileiro, iniciando o que até então foi o maior surto de febre amarela silvestre das últimas décadas no país, com 779 casos humanos confirmados no período de monitoramento de julho de 2016 a junho de 2017 e 262 óbitos (letalidade de 33,6%). Os quatro estados da região Sudeste apresentaram o maior número de casos: Minas Gerais (MG), Espírito Santo (ES), São Paulo (SP) e Rio de Janeiro (RJ). Essa região possui grandes centros urbanos, com população que, em grande parte, não estava imunizada contra a doença e muitas localidades com alta densidade de infestação pelo *Aedes aegypti* ^3 , 4^.

No período de julho de 2017 a 16 de maio de 2018, ocorreu novo surto de maior magnitude no país, com 1.266 casos humanos confirmados e 415 óbitos (letalidade de 32,8%), predominando MG, SP e RJ^6^. Foram também confirmados óbitos em primatas não humanos em outros estados brasileiros, levantando a relevância da adoção de medidas para reduzir o risco de reurbanização da doença, com ampliação das áreas de vacinação recomendada e início das campanhas com aplicação da dose fracionada^3 , 7^.

Em 2018, segundo dados da Secretaria de Estado de Saúde do Rio de Janeiro (SES/RJ) atualizados em 5 de julho de 2018, havia neste estado 264 casos confirmados, em 32 municípios, com 85 óbitos (32,2%); 13 localidades tinham epizootias confirmadas, com morte de macacos^8^.

Historicamente, no RJ, o Instituto Estadual de Infectologia São Sebastião (IEISS), uma referência para atendimento de doenças infecciosas, foi integrado à área física do Hospital Federal dos Servidores do Estado (HFSE) em 20 de agosto de 2012. O HFSE, por sua vez, é um hospital geral, federal e de ensino, que integra a Rede de Vigilância Epidemiológica Hospitalar de Interesse Nacional^9^. Nesse contexto do surto de febre amarela, essa estrutura aqui denominada HFSE/IEISS teve importante papel na atenção aos casos graves, internando 52 pacientes confirmados de 11 de março de 2017 a 15 de junho de 2018. O objetivo deste estudo é descrever o perfil clínico-epidemiológico desses casos e os fatores associados ao óbito.

## MÉTODOS

Realizou-se este estudo em um hospital referência para doenças infecciosas do RJ que conta com um serviço de epidemiologia integrante da rede nacional, o qual realiza busca ativa dos casos atendidos na unidade^9^. O RJ tem como limites os estados de MG, ES e SP, além do Oceano Atlântico. Ocupa 43.781,588 km^2^distribuídos em 92 municípios, com 16.718.956 habitantes em 2017, sendo 96,7% em área urbana^10^. Cerca de 30% da área do estado é ocupada por Mata Atlântica^11^.

De 11 de março de 2017 a 15 de junho de 2018, foram notificados no HFSE/IEISS 68 casos suspeitos da doença, de acordo com a definição para fins de vigilância epidemiológica: “indivíduo com quadro febril agudo (até sete dias), de início súbito, acompanhado de icterícia ou manifestações hemorrágicas, residente em (ou procedente de) área de risco para febre amarela ou de locais com ocorrência de epizootia confirmada em primatas não humanos ou isolamento de vírus em mosquitos vetores, nos últimos 15 dias, não vacinado contra febre amarela ou com estado vacinal ignorado”^1 , 2^. Desses, 52 foram confirmados e internados no hospital (43 em 2018), constituindo a amostra do estudo. O critério de confirmação foi o exame de reação em cadeia da polimerase (PCR) detectável em 51 casos, enquanto o outro foi confirmado por vínculo epidemiológico^1 , 2^.

O delineamento foi observacional, retrospectivo, com análise de bases de dados secundários da vigilância epidemiológica no HFSE a partir do Sistema de Informação de Agravos de Notificação (Sinan) e coleta complementar de dados dos prontuários clínicos, realizada pela equipe do serviço de epidemiologia, durante o processo de investigação epidemiológica dos casos. Analisaram-se variáveis demográficas, epidemiológicas, clínicas e laboratoriais em banco de dados construído em planilha eletrônica, exportado a partir do Sinan e complementado pela digitação de variáveis adicionais às existentes na ficha de investigação de febre amarela^2 , 12^, correspondentes a sinais, sintomas e exames laboratoriais (detalhadas nas Tabela 1 e Tabela 2).

**Tabela 1:** Perfil dos 52 casos confirmados de febre amarela internados no HFSE/IEISS. Rio de Janeiro, RJ, Brasil, 11 de março de 2017 a 15 de junho de 2018.

Dados clínicos	f	%
Sexo masculino	45	86,5
Faixa etária (anos)		
13–34	12	23,1
35–49	14	26,9
50–59	13	25,0
60–75	13	25,0
Febre	47	90,4
Icterícia	45	86,5
Náuseas/vômitos	36	69,2
Cefaleia	28	53,8
Alteração de excreção renal (oligúria, anúria ou ambas)	28	53,8
Hemorragia	26	50,0
Dor abdominal	25	48,1
Mialgia	17	32,7
Prostração/astenia	14	26,9
Diarreia	9	17,3
Colúria	9	17,3
Artralgia	6	11,5
Convulsões	6	11,5
Dor retro-orbitária	2	3,8
Edema articular	2	3,8
Exantema	1	1,9
Sinal de Faget	1	1,9
Hiperemia conjuntival	1	1,9
Plaquetopenia < 100.000/mm^3^	45	86,5
Leucopenia	34	65,4
Comorbidade (pelo menos uma)	20	38,5
Hipertensão arterial	10	19,2
Etilismo	9	17,3
Diabetes	5	9,6
Tabagismo	4	7,7
Esteatose hepática	2	3,8
Artrite reumatoide	2	3,8
Antecedentes epidemiológicos e conclusão da investigação		
Presença de *Aedes aegypti* na região	51	98,1
Epizootias no município de residência do caso	35	67,3
Vacinado contra febre amarela	9	17,3
Classificação final como febre amarela silvestre	52	100,0
Critério de confirmação laboratorial (PCR)	51	98,1
Caso autóctone do município de residência	34	65,4
Doença ocupacional	10	19,2
Evolução		
Óbito por febre amarela silvestre	21	40,4
Cura	31	59,6

HFSE/IEISS: Hospital Federal dos Servidores do Estado e Instituto Estadual de Infectologia São Sebastião; PCR: reação em cadeia da polimerase

**Tabela 2:** Distribuição dos valores séricos de alguns exames segundo a evolução dos casos confirmados de febre amarela internados no HFSE/IEISS. Rio de Janeiro, RJ, Brasil, 11 de março de 2017 a 15 de junho de 2018.

Valores séricos	Total de casos		Óbito		Não óbito		Teste de Mann-Whitney (p)
		
	n	Mediana (mínimo–máximo)	n	Mediana (mínimo–máximo)	n	Mediana (mínimo–máximo)	
AST máxima (U/L)	50	4.833 (69–12.970)	19	8.460 (1.803–12.970)	31	1.820 (69–10.900)	0,000
ALT máxima (U/L)	51	2.920 (86–8.064)	20	5.379 (1.943–8.064)	31	1.581(86–5.428)	0,000
BT máxima (mg/dL)	52	5,62 (0,70–38,00)	21	7,00 (2,00–38,00)	31	4,23 (0,70–29,00)	0,063
BD máxima (mg/dL)	51	4,03 (0,09–20,00)	21	5,00 (2,00–20,00)	30	3,07 (0,09–17,00)	0,043
Ureia máxima (mg/dL)	49	54 (15–180)	20	93 (45–161)	29	36 (15–180)	0,000
Creatinina máxima (mg/dL)	52	1,72 (0,7–17,5)	21	3,2 (0,80–11,7)	31	1,2 (0,7–17,5)	0,000
Plaquetemia mínima (por mm^3^)	52	50.000 (10.000–232.000)	21	38.000 (10.000–26.000)	31	68.000 (15.000–232.000)	0,008
Hematócrito mínimo (%)	50	36,0 (12,9–51,6)	20	29,5 (13,2–51,6)	30	38,5 (12,9–50,0)	0,081

HFSE/IEISS: Hospital Federal dos Servidores do Estado e Instituto Estadual de Infectologia São Sebastião; AST: aspartato aminotransferase; ALT: alanina aminotransferase; BT: bilirrubina total; BD: bilirrubina direta.

A análise estatística foi realizada no programa SPSS v.18, envolvendo análise descritiva bivariada e múltipla por regressão logística para estudo da associação das variáveis independentes com o desfecho óbito. Para estimar a chance de óbito, foram usadas *odds ratio* (OR) e seus respectivos intervalos de confiança de 95% (IC95%).

Na análise bivariada, utilizou-se o teste qui-quadrado de Pearson ou o teste exato de Fisher para as variáveis categóricas, e o teste de Mann-Whitney para as variáveis contínuas. Considerou-se estatisticamente significativo o valor de p < 0,05. Para identificar o ponto de corte para variáveis numéricas correspondentes aos resultados de exames laboratoriais, utilizou-se a análise de agrupamentos por *K-means* , que consiste na subdivisão de um conjunto de dados em um número previamente escolhido de agrupamentos. Esse tipo de análise é utilizado quando não temos hipótese *a priori* sobre a estrutura ou comportamento dos dados e constitui um agrupamento não hierárquico por repartição. Optou-se por subdividir os dados de cada variável contínua em dois agrupamentos, utilizando como ponto de partida a identificação dos valores mínimos e máximos registrados. Ao final do processo, obteve-se um ponto de corte aproximado entre a média do valor máximo do primeiro agrupamento e o mínimo do agrupamento seguinte.

Para a regressão logística, foram incluídas as variáveis que apresentaram associação com o óbito na análise bivariada prévia, com p < 0,10, e aquelas consideradas de importância clínica (como idade, sexo, comorbidade e dor abdominal) ou de interesse (como ano de notificação). O teste de Wald foi usado para testar a significância das variáveis independentes incluídas nos modelos estudados. Para a análise do ajuste global, utilizou-se o teste da razão de verossimilhança ( *-2 log likelihood* ). Foi analisada a concordância entre as probabilidades preditas pelos modelos e as respostas observadas, e usado o teste de Hosmer-Lemeshow.

Estudo incluído em projeto aprovado pelo Comitê de Ética em Pesquisa do HFSE (parecer CEP-HFSE 000.534 de 14 de julho de 2014).

## RESULTADOS

A Figura 1 apresenta a distribuição temporal dos casos por semana epidemiológica da notificação; 35 (78,8%) ocorreram de janeiro a março de 2018. Desses, 45 (86,5%) eram homens e sete (13,5%) mulheres, com idade entre 13 e 75 anos (mediana de 49,5 anos). A raça ou cor de pele autorreferida foi branca em 29 (55,8%), parda em 15 (28,8%), preta em sete (13,5%), e amarela em um caso (1,9%); 27 casos tinham no máximo ensino fundamental incompleto (73,0%; excluídos 15 com escolaridade ignorada).

**Figura 1:** Distribuição segundo semana epidemiológica da notificação dos casos confirmados de febre amarela internados no Hospital Federal dos Servidores do Estado/Instituto Estadual de Infectologia São Sebastião. Rio de Janeiro, RJ, Brasil, 11 de março de 2017 a 15 de junho de 2018.

Houve casos de 18 municípios do RJ, sendo 50 residentes e dois turistas chilenos. Em 2018, 21 casos foram oriundos da região turística da Costa Verde, sendo 18 da Baía de Ilha Grande (16 residentes e os dois turistas chilenos). A zona de residência ou estadia era rural em 32 (61,5%), urbana em 11 (21,2%), periurbana em dois (3,8%) e ignorada em sete (13,5%). A ocupação predominante era trabalhador rural (21 casos; 40,4%); três casos realizavam atividades turísticas (dois turistas e um guia).

A Tabela 1 apresenta o perfil dos casos segundo variáveis clínicas, epidemiológicas e conclusão da investigação epidemiológica. Os sinais e sintomas mais frequentes foram: febre (90,4%), icterícia (86,5%) e náuseas e vômitos (69,2%). Alterações de excreção renal (oligúria ou anúria) ocorreram em 53,8%, hemorragias em 50,0% e dor abdominal em 48,1%. Plaquetopenia < 100.000/mm^3^ocorreu em 86,5% e leucopenia em 65,4%.

Em 38,5% dos casos, descreveu-se pelo menos uma comorbidade, predominando a hipertensão arterial sistêmica (19,2% dos casos), etilismo (17,3%) e diabetes (9,6%). Em 98,1% relatou-se presença de *Aedes aegypti* em área urbana por onde o paciente circulou. Epizootias em primatas não humanos foram relatadas no município de residência de 67,3%.

Em nove casos (17,5%), havia história de vacinação prévia contra febre amarela; o intervalo entre a data da vacina e o início dos sintomas foi de até quatro dias em cinco deles; entre 11 e 12 dias em dois; sete anos em um; e um caso foi vacinado no terceiro dia do início dos sintomas. Nos casos com história de vacinação prévia recente, houve comprovação do vírus selvagem por sequenciamento, afastando-se evento adverso pós-vacinação.

Em 65,4% dos casos, a transmissão foi autóctone do município de residência; nos demais, indeterminada, por haver deslocamento para outros municípios do próprio RJ. Em 19,2%, o vínculo ocupacional foi bem caracterizado ( Tabela 1 ). Quanto à evolução, 21 casos (40,4%) foram a óbito (19 em 2018 e 2 em 2017).

O retardo entre o início dos sintomas e a admissão no HFSE/IEISS variou de um a 15 dias, com mediana de cinco, semelhante entre os casos que faleceram e os que não faleceram. O tempo entre início dos sintomas e o óbito variou de quatro a 22 dias, com mediana de nove. O tempo de permanência no hospital variou de um a 56 dias, com mediana de 10, sem diferença significativa entre óbitos (1 a 26 dias; mediana = 11) e sobreviventes (3 a 56 dias; mediana = 10). O intervalo entre a admissão e a notificação do caso à vigilância epidemiológica variou entre zero e três dias, com mediana de um; 86,5% foram notificados em até 24 horas da admissão.

A Tabela 2 apresenta a distribuição dos valores séricos máximos de aspartato aminotransferase (AST), alanina aminotransferase (ALT), bilirrubina total (BT), bilirrubina direta (BD), ureia, creatinina e o menor valor de plaquetemia e de hematócrito para o conjunto dos casos e segundo o desfecho óbito. Houve associação significativa entre maiores valores de AST, ALT, BD, ureia e creatinina e óbito, assim como entre menores valores de plaquetemia e óbito.

Segundo a Tabela 3 , a presença dos seguintes sinais e sintomas esteve significativamente associada à maior chance de óbito: icterícia, hemorragia, alterações de excreção renal e convulsões. O relato de leucopenia associou-se significativamente a uma menor letalidade. Na Tabela 3 , os pontos de corte para AST, ALT, ureia e creatinina foram identificados pela análise por *K-means* , que definiu, para cada uma, dois clusters (1 e 2), com seus valores mínimos e máximos descritos a seguir: AST – cluster 1 (69 e 5.680 U/L) e 2 (6.260 e 12.970 U/L); ALT – 1 (86 e 3.846 U/L) e 2 (4.160 e 8.064 U/L); ureia – 1 (15 e 93 mg/dL) e 2 (110 e 180 mg/dL); creatinina – 1 (0,7 e 5,7 mg/dL) e 2 (7 e 17,5 mg/dL). Os seguintes pontos de corte de resultados laboratoriais séricos se associaram significativamente à maior chance de óbito: AST > 6.000 U/L; ALT > 4.000 U/L, ureia > 100 mg/dL e creatinina > 6 mg/dL. Um subgrupo de 20 casos com valores máximos de AST abaixo de 3.000 U/L teve letalidade de 5,0% (um óbito), e outro de 18 casos com ALT abaixo de 3.500 U/L apresentou letalidade de 5,6% (um óbito). Não foi observada associação entre o óbito e as variáveis sexo, faixa etária, raça ou cor de pele, escolaridade, ano de notificação, retardo entre início dos sintomas e internação no hospital, dor abdominal e comorbidade.

**Tabela 3:** Letalidade e estimativa da chance de óbito segundo algumas variáveis dos casos confirmados de febre amarela internados no HFSE/IEISS. Rio de Janeiro, RJ, Brasil, 11 de março de 2017 a 15 de junho de 2018.

Variáveis clínicas e exames laboratoriais séricos	Presente			Ausente			Teste qui-quadrado (p)	*Odds ratio*	IC95%
	
	Casos	Óbitos		Casos	Óbitos				
			
	f	f	%	f	f	%			
Icterícia	45	21	46,7	7	0	0,0	0,033^a^	NA	NA
Hemorragia	26	17	65,4	26	4	15,4	0,000	10,389	2,728–39,560
Alteração da excreção renal	28	19	67,9	24	2	8,3	0,000	23,222	4,457–120,989
Convulsões	6	5	83,3	46	16	34,8	0,034^a^	9,375	1,007–87,284
Leucopenia	34	10	29,4	18	11	61,1	0,027	0,265	0,079–0,881
AST^b^máxima > 6.000 U/L	20	14	70,0	30	5	16,7	0,000	11,667	3,009–45,239
ALT^c^máxima > 4.000 U/L	16	13	81,3	35	7	20,0	0,000	17,333	3,852–77,994
Ureia máxima > 100 mg/dL	12	9	75,0	37	11	29,7	0,006	7,091	1,607–31,296
Creatinina máxima > 6 mg/dL	12	8	66,7	40	13	32,5	0,034	4,154	1,055–16,355

HFSE/IEISS: Hospital Federal dos Servidores do Estado e Instituto Estadual de Infectologia São Sebastião; NA: não avaliado

^a^Teste exato de Fisher

^b^Aspartato aminotransferase

^c^Alanina aminotransferase

Na análise múltipla, alguns modelos logísticos foram estudados. Partiu-se de um modelo saturado com sexo, idade, comorbidades, hemorragia, alteração de excreção renal, leucopenia e variáveis laboratoriais contínuas (bilirrubinas, AST, ALT, ureia, creatinina e plaquetemia), pelo método *stepwise* . Permaneceram no modelo 1 ( Tabela 4 ), com associação significativa com maior chance de óbito, a presença de alteração de excreção renal e valores crescentes de ALT. Os modelos 2 e 3 foram construídos pelo método *enter* , optando-se por manter a AST, apesar de ela não ter alcançado significância estatística. Neles, a associação da alteração de excreção renal com maior chance de óbito permaneceu. Adicionalmente, no modelo 2, no qual as transaminases foram categorizadas em faixas identificadas na análise bivariada (ALT ≤ 4.000 U/L e ALT > 4.000 U/L; AST ≤ 6.000 U/L e AST > 6.000 U/L), observou-se associação significativa entre maior chance de óbito e ALT > 4.000 U/L, e significância limítrofe para AST > 6.000 U/L. No modelo 3, as transaminases estão em escala que varia a cada 1.000 U/L. A cada aumento de 1.000 U/L no valor da ALT, há um aumento significativo de 137% na chance de óbito, e um aumento de significância limítrofe no caso da AST. Os modelos 1, 2 e 3 apresentaram concordâncias satisfatórias (85,7%, 88,0% e 90,0% respectivamente). O teste de Hosmer-Lemeshow confirmou ajuste satisfatório para os três, obtendo valores não significativos para a diferença entre as frequências observadas e esperadas dentro de cada decil de probabilidade estimada de óbito (p = 0,490, p = 0,179 e p = 0,799, respectivamente).

**Tabela 4:** Análise múltipla por regressão logística dos fatores associados ao óbito nos casos confirmados de febre amarela internados no HFSE/IEISS. Rio de Janeiro, RJ, Brasil, 11 de março de 2017 a 15 de junho de 2018.

Variável	Parâmetro	Erro-padrão	p	*Odds ratio*	IC95%
Modelo 1 (concordância: 85,7%)^a^					
Intercepto	-6,504	2,063	0	-	-
Alteração de excreção renal (referência: ausente)	3,314	1,29	0,01	27,501	2,193–344,82
ALT (contínua)	0,001	0	0,004	1,001	1–1,002
Modelo 2 (concordância: 88,0%)^b^					
Intercepto	-4,48	1,401	0,001	-	-
Alteração de excreção renal (referência: ausente)	3,433	1,261	0,006	30,977	2,618–366,563
ALT > 4.000 U/L (referência: ≤ 4.000 U/L)	2,811	1,165	0,016	16,633	1,696–163,122
AST > 6.000 U/L (referência: ≤ 6.000 U/L)	1,7	0,926	0,066	5,471	0,891–33,582
Modelo 3 (concordância: 90,0%)^c^					
Intercepto	-8,101	2,773	0,003	-	-
Alteração de excreção renal (referência: ausente)	3,218	1,413	0,023	24,981	1,567–398,245
ALT 1.000 (variação a cada 1.000 U/L)	0,862	0,399	0,031	2,367	1,083–5,177
AST 1.000 (variação a cada 1.000 U/L)	0,394	0,226	0,081	1,483	0,953–2,310

HFSE/IEISS: Hospital Federal dos Servidores do Estado e Instituto Estadual de Infectologia São Sebastião

^a^Modelo 1: *stepwise forward* a partir de sexo, idade, comorbidade, alteração da excreção renal, aspartato aminotransferase (AST), alanina aminotransferase (ALT), bilirrubina direta, ureia, creatinina e leucopenia.

^b^Modelo 2: *enter* com ALT e AST categóricas.

^c^Modelo 3: *enter* com ALT e AST em escala de 1.000 unidades de variação.

Quanto ao tratamento, além das medidas de suporte intensivo, a troca plasmática de alto volume com objetivo de remoção de citocinas plasmáticas e moléculas de adesão e reposição de fatores de coagulação foi realizada em 12 pacientes (23,1%), ocorrendo 5 óbitos (41,7%); a letalidade nos demais casos foi de 40,0%. Hemodiálise foi realizada em 21 casos (40,4%), com 16 óbitos (letalidade de 76,2% *versus* 16,1% nos demais; OR = 16,6; IC95% 4,2–66,2). Em modelo multivariado controlando transaminases, ureia, creatinina e plaquetemia, a associação entre hemodiálise e óbito perdeu a significância estatística (OR = 8,3; IC95% 0,6–119,2).

## DISCUSSÃO

Este estudo apresentou o perfil de 52 casos confirmados de febre amarela internados no HFSE/IEISS, que foi responsável pela hospitalização de 17,9% dos casos confirmados no estado do Rio de Janeiro em 2017 e 2018. Em 2017, a SES/RJ confirmou 27 casos com 9 óbitos^13^; em 2018, 264 casos e 85 óbitos^8^. A distribuição temporal dos casos internados no hospital acompanhou a dinâmica do surto no estado.

O predomínio do sexo masculino é coerente com a literatura^14^. A mediana de idade (49,5 anos) foi maior do que a de outros estudos: 26 e 27,5 anos^15 , 16^. Estudos de casos na região extra-amazônica têm relatado distribuições etárias mais próximas à deste estudo, como mediana de 36 anos^14^e média de 46,7 anos^18^. As diferenças de distribuição dos casos de febre amarela silvestre por idade e sexo têm variado de acordo com o grau de exposição à mata^14^.

A zona de residência foi considerada rural em 61,5% dos casos, percentual próximo ao de outro estudo^14^. Quanto à ocupação, 40,4% eram trabalhadores rurais com evidente exposição ao ciclo silvestre; outros estudos relatam percentuais próximos ou maiores, de 41,7%^17^a 79,2%^14 , 16 , 18^. A contribuição da atividade turística tem sido descrita^14 , 17^. Relato recente mostrou 10 casos de febre amarela em viajantes no Brasil, com quatro óbitos; três deles eram chilenos, dos quais dois estão incluídos no presente estudo^21^.

Com relação ao estado vacinal, estudo sobre 281 casos confirmados de 1999 a 2003, dos quais 37,4% eram da Amazônia, relatou que 90,8% eram não vacinados e que os 9,2% restantes receberam apenas uma dose^14^. Naquele estudo, o indivíduo que recebera a vacina até dez dias antes do início dos sintomas foi considerado não vacinado^4^. Por esse critério, apenas três casos do presente estudo seriam considerados efetivamente vacinados. No contexto deste estudo, tornou-se relevante a exclusão do diagnóstico diferencial com evento adverso pós-vacinação nos casos com história de vacinação recente.

Estima-se que pelo menos 90% dos casos de febre amarela sejam assintomáticos ou oligossintomáticos, e que 10% sejam formas graves associadas a letalidade alta.^5^Sintomas gerais comumente relatados são febre (85%^18^a 94,4%^15 , 19^), cefaleia (70,3%^18^a 83,3%^15 , 19^), mialgia (27,8%^19^a 76,5%^18^) e vômitos (69,4%^19^a 75,8%^15^), em percentuais próximos ao deste estudo – à exceção de cefaleia, que foi menor (53,8%).

Entretanto, o percentual de 86,5% de icterícia deste estudo é maior do que o relatado por outros, que variam de 35,5% a 69,1%^15 , 18 , 19^. Também nosso percentual de alterações de excreção renal (53,8%) é maior do que relatado por outros autores: 16,7%^19^a 36,7%^15^. Esse último estudo^15^relatou 46,4% de manifestações hemorrágicas, percentual próximo ao nosso (50%).

Segundo a literatura, aproximadamente 15% dos casos desenvolvem doença visceral com icterícia. Nessa situação, as taxas de letalidade variam de 20% a 50%^4 , 22^. A letalidade observada na África tem sido em torno de 20%, em geral menor do que na América do Sul (40%–60%)^4^. As letalidades relatadas em pacientes com doença viscerotrópica ou pós-vacinal grave são elevadas, acima de 50%, mesmo em centros desenvolvidos e com terapia intensiva^22^.

No Brasil, a letalidade dos casos graves oscila entre 40 e 60%^5 , 14^. Há estudos com letalidades menores, porém com perfil de gravidade diferente. Um surto no estado do Maranhão, com 40,0% de casos assintomáticos, relatou 17,5% de letalidade^20^. Recente estudo em MG encontrou letalidade de 13,5%, porém a frequência relatada de icterícia foi 35,5%^18^, inferior à do presente estudo (86,5%). Outro surto em MG, no qual 69,4% dos casos foram hospitalizados, encontrou 33,3% de letalidade; em 19 casos classificados como graves (com manifestações hemorrágicas, insuficiência hepatorrenal, prostração intensa ou coma), a letalidade foi de 83,3%^19^.

Considerando apenas o critério icterícia, 86,5% os casos deste estudo já estariam no mínimo na forma clínica moderada ou grave^1 , 4^. Outro estudo descreveu 70% de forma moderada a grave, 69,1% de icterícia e 44,2% de óbitos^15^.

Não foi observada associação entre óbito e sexo, faixa etária, raça ou cor de pele, escolaridade, ano de notificação, retardo entre início dos sintomas e internação no hospital, dor abdominal e comorbidade. Estudo sobre casos confirmados no Brasil de 2000 a 2012^16^encontrou letalidade maior no sexo masculino (49,6%) do que no feminino (39,7%), porém a diferença não foi significativa. Estudo de período anterior (1999–2002) encontrou associação significativa da maior chance de óbito como o sexo masculino e a faixa etária acima de 40 anos, mas na análise múltipla sexo e idade perderam a significância^15^.

A mediana do intervalo entre o início dos sintomas e a admissão no HFSE/IESS (cinco dias) foi um pouco maior do que a de outro estudo (três dias)^19^. Ambos não encontraram diferenças entre esse intervalo e o óbito, achado concordante com pelo menos outro estudo^18^. No presente estudo, o primeiro atendimento ocorreu em outras unidades, com encaminhamento posterior para o HFSE/IEISS.

Estudo de MG^18^relatou tempo de permanência menor do que o deste estudo, também sem diferença segundo o desfecho (média = 5,70 dias e DP = 3,78 dias nos casos de óbito; média = 5,02 dias e DP = 5,88 dias nos sobreviventes). Outro estudo relatou uma mediana do tempo de internação até o óbito de oito dias^19^, menor do que a nossa. A gravidade dos casos do presente estudo e a utilização de terapia intensiva, hemodiálise e troca plasmática de alto volume são alguns fatores possivelmente relacionados ao maior tempo de permanência e também ao alto custo da assistência.

De forma geral, os valores séricos de transaminases, bilirrubinas, ureia e creatinina deste estudo foram maiores do que os relatados na literatura. Estudo com 36 casos, com 91,7% de formas graves e moderadas^19^, descreveu para a AST uma variação de 47 a 7.500 U/L (média = 1.027,8); ALT, 26 a 6.600 U/L (média = 815,8); BD, 0,2 a 12,4 mg/dL (média = 3,2); ureia, 10 a 175 mg/dL (média = 56,5); creatinina, 0,6 a 13 mg/dL (média = 2,9). Outro estudo^15^, com 251 casos e 70,4% de forma moderada a grave, relatou medianas de transaminases menores, embora os valores máximos fossem maiores que os nossos: AST, mediana de 1.096 U/L (variação de 13 a 43.460); ALT, mediana de 957 U/L (9 a 15.250); BD, mediana de 4,7 mg/dL (0,4 a 77,0). Entretanto, para ureia e creatinina, seus valores foram maiores: ureia, mediana de 106 mg/dL (11 a 328); creatinina, mediana de 3,6 mg/dL (1,0 a 19,0).

Tanto no presente estudo como nos dois outros^15 , 19^, de forma geral as elevações da AST ultrapassaram as da ALT, o que é descrito na literatura^5 , 23^presumivelmente devido ao efeito citopático do vírus no miocárdio e músculos esqueléticos; a ALT é mais específica para doença hepática do que a AST. Quanto ao aumento das bilirrubinas, ocorreu sobretudo, pelo aumento da fração direta, coerente com a literatura^5^.

Neste estudo, encontrou-se associação significativa entre maiores valores de AST, ALT, BD, ureia e creatinina e óbito, assim como entre menores valores de plaquetemia e óbito. Na análise múltipla, apenas a alteração de excreção renal e a ALT permaneceram significativamente associadas à maior chance de óbito; a AST manteve uma associação com p limítrofe. Outro estudo^15^encontrou associação com maior chance de óbito para sexo masculino, idade > 40 anos, icterícia, AST > 1.200 U/L, ALT > 1.500 U/L, BT > 7 mg/dL, BD > 5 mg/dL e ureia > 100 mg/dL. Porém, na análise múltipla apenas permaneceram as associações com icterícia e AST > 1.200 U/L.

Não há tratamento específico até o momento. Recomenda-se a internação das formas graves em hospitais com suporte de terapia intensiva e diálise^1 , 2 , 23^. Alguns antivirais têm sido avaliados, como ribavirina, tiazofurina, carboxamida e compostos pirazolínicos^4 , 22^, e mais recentemente o sofosbuvir^7^. Os eventos terminais catastróficos associados aos casos graves, possivelmente relacionados a uma síndrome de resposta inflamatória sistêmica, representam um campo potencial para avaliação do papel de intervenções como a troca plasmática de alto volume^7^, que foi realizada na instituição. Essa utilização ocorreu a partir da lógica das diretrizes de manejo da hepatite fulminante; o protocolo usado foi aquele referido por Larsen et al.^24^, adaptado para as circunstâncias do surto de febre amarela.

O transplante hepático na hepatite fulminante da febre amarela foi realizado pela primeira vez em dezembro de 2017 em São Paulo, não tendo seu papel bem estabelecido^7 , 23 , 25^. No presente estudo, um caso foi transferido para realização de transplante hepático em outra unidade, evoluindo para óbito em menos de 24 horas após a transferência, durante o período transoperatório (óbito incluído neste estudo).

A reemergência da febre amarela fora da região amazônica atingiu regiões próximas a grandes centros urbanos e com população que, em grande parte, não estava imunizada. Nos últimos dois anos, a expansão da doença, principalmente pela região Sudeste, resultou na maior epidemia das últimas décadas. As causas dessa expansão ainda não estão bem esclarecidas; fatores ambientais e alterações do próprio vírus têm sido investigados^7^. Em 2017, identificou-se uma mutação nas enzimas de replicação viral, mas não se conhece ainda seu impacto^26^. Estudo recente considera que o vírus da febre amarela responsável pelos surtos de 2016 a 2018 no Sudeste brasileiro pertença a uma linhagem monofilética (BR-YFV_2016/18), provavelmente vinda da região Centro-Oeste para a Sudeste. A sua persistência no Sudeste sugere que essa região apresenta condições ecológicas e climáticas adequadas para a manutenção viral nos períodos epidêmicos e interepidêmicos^27^. Estação chuvosa prolongada associada a abundância de vetores, desmatamento para agropecuária, transmissão epizoótica e locomoção de indivíduos em período virêmico são fatores citados^4 , 28^.

A alta infestação por *Aedes aegypti* em diversos centros urbanos dessas regiões, o aumento do número de casos de febre amarela silvestre e a baixa cobertura vacinal são fatores que favorecem o risco da reurbanização da doença. Os recentes surtos de febre amarela silvestre a partir de dezembro de 2016, atingindo os estados de MG, ES, RJ e SP, e a ocorrência de óbitos confirmados em macacos em outros estados levantaram novamente a relevância da adoção de medidas para reduzir o risco de reurbanização, com alteração nas recomendações de vacinação, incluindo a ampliação das áreas de recomendação da vacina e o início das campanhas com aplicação da dose fracionada^3 , 7^.

Nesse contexto, este estudo apresentou a experiência de um hospital que contribuiu de forma expressiva para a assistência aos casos graves de febre amarela do estado. A gravidade da série está representada pelo elevado percentual de icterícia (86,5%), alterações de excreção renal (53,8%) e hemorragias (50,0%), além das elevadas medianas de transaminases séricas observadas. A atenção a esses casos implicou em uma estrutura de complexidade alta, adaptada para responder a uma situação emergencial, que representou um grande desafio para a equipe de saúde. O maior conhecimento do perfil desses casos, sobretudo dos fatores associados à maior chance de óbito, pode colaborar para a discussão do papel de intervenções terapêuticas como a troca plasmática de alta volume. As limitações deste estudo se relacionam principalmente à qualidade da informação oriunda da vigilância epidemiológica, o que foi minimizado pela revisão da investigação e complementação de dados com os prontuários durante o processo de investigação epidemiológica e encerramento dos casos, que faz parte da rotina do serviço de epidemiologia^9^. O percentual de informações ignoradas foi inferior a 5% para a maioria das variáveis analisadas, à exceção da escolaridade (15,0%) e ureia máxima (7,7%), e muito menores do que em outros estudos^15^.

A vigilância epidemiológica ativa contribuiu com a notificação ágil às autoridades de saúde, o monitoramento da coleta das amostras para confirmação dos casos e a manutenção de um sistema de informações que, mesmo durante um surto, foi capaz de subsidiar este estudo. O estudo contribuiu para o conhecimento do perfil dos casos de febre amarela no HFSE/IEISS, que internou 17,9% dos casos confirmados no RJ nesse período, com perfil de gravidade alta. Os principais preditores de óbito foram a alteração da excreção renal e a elevação sérica de transaminases, sobretudo ALT. A letalidade elevada reforça a necessidade de diagnóstico e tratamento precoces, e a importância de intensificar a vacinação.
